# Plasma–Nanomedicine Synergistic Therapy for Brain Diseases: Current Status, Applications, and Challenges

**DOI:** 10.3390/antiox15020166

**Published:** 2026-01-26

**Authors:** Shun-Lian Li, Qiao Li, Jun-Ze Deng, Zhen-Long Zhang, Miao Qi, Xiu-Hua Luo, Yudan Zhang, Qing-Yan Ma, Feng Zhu, Xian-Cang Ma, Dao-Cheng Wu, Shuo Zhang

**Affiliations:** 1 Center for Brain Science & Department of Psychiatry & Center for Translational Medicine, The First Affiliated Hospital of Xi’an Jiaotong University, Xi’an 710061, China; 15887367230@163.com (S.-L.L.); 18220606851@163.com (Q.L.); junze0825@163.com (J.-Z.D.); 15703028010@163.com (X.-H.L.); zhangyudan@xjtufh.edu.cn (Y.Z.); mqy910413@163.com (Q.-Y.M.); zhufeng1982@xjtu.edu.cn (F.Z.); maxiancang@163.com (X.-C.M.); 2 Clinical Medical College, Shaanxi University of Chinese Medicine, Xianyang 712046, China; 3 State Key Laboratory for Manufacture Systems Engineering, Xi’an Jiaotong University, Xi’an 710049, China; zhangzhenlongnx@126.com (Z.-L.Z.); qimiao@stu.xjtu.edu.cn (M.Q.); 4 The Key Laboratory of Biomedical Information Engineering of Ministry of Education, School of Life Science and Technology, Xi’an Jiaotong University, Xi’an 710049, China

**Keywords:** CAP, nanomedicine, brain diseases, synergistic therapy, neurodegenerative diseases

## Abstract

Brain diseases such as ischaemic stroke, Alzheimer’s disease (AD), and glioma were characterized by high mortality and disability rate, and oxidative stress remains a major obstacle in treatment. Plasma–nanomedicine synergistic treatment technology provides a very attractive treatment strategy based on complementarity. This technology integrates cold atmospheric plasma (CAP) with nanomedicine. CAP produces active substances that regulate oxidative stress, while nanomedicine is specially designed for targeted delivery, controlled release, and microenvironmentally responsive activation of therapeutic agents. This integration generates new therapeutic functions and significantly improves the overall therapeutic effect. Despite the broad prospects of this emerging technology, researchers in the fields of medicine, physics, or pharmacy have not yet paid much attention to it. To fill this research gap, this review describes the physicochemical properties and biological effects of CAP and summarizes the latest advances in plasma nanomedicine strategies in the field of brain disease intervention, and reviews the four major nanomedical categories—metal-based, inorganic non-metallic, polymer-based and hydrogel systems—and their clinical applications in the treatment of brain tumors, strokes and neurodegenerative diseases in conjunction with CAP. Finally, we highlight a number of key challenges—limited resources of special CAP equipment, incomplete understanding of the mechanism, obstacles to transformation application—and put forward the future research direction to promote the development of accurate, safe, and clinical transformation value plasma–nanomedicine therapy for brain diseases.

## 1. Introduction

Neurological diseases, including AD, ischaemic stroke, Parkinson’s disease (PD), and glioma, represent major global public health challenges due to high mortality, long-term disability, and substantial socioeconomic burden on patients, families, and healthcare systems worldwide. According to the World Health Organization (WHO), more than 3 billion people—approximately 43.1% of the global population—are affected by neurological disorders [1]. Despite intensive research efforts, effective treatment remains constrained by two closely interrelated bottlenecks: the restrictive nature of the blood–brain barrier (BBB) and the absence of precise strategies for regulating oxidative stress, a central pathological driver shared by most neurological diseases [2,3]. In response, this review highlights an emerging interdisciplinary strategy that integrates plasma technology with nanomedicine. By leveraging their complementary strengths, plasma confers enhanced bioactivity and additional physical therapeutic modalities to nanomaterials, while nanomedicine enables targeted BBB penetration and stimulus-responsive drug release, together establishing a new paradigm for precise and minimally invasive treatment of brain diseases.

The primary objective of this review is to summarize recent advances in plasma–nanomedicine synergistic therapy, elucidate its fundamental physicochemical mechanisms, and address critical gaps hindering further development. Specifically, this review aims to (1) clarify the interaction mechanisms between CAP and nanocarriers, including plasma-induced activation of nanomaterials and nanomaterial-mediated delivery of plasma-generated bioactive species; (2) extend the scope of application beyond well-studied brain diseases such as glioma, AD, and ischaemic stroke to understudied yet clinically relevant conditions, including depression and vascular cognitive impairment, which are strongly associated with oxidative stress dysregulation and neural circuit dysfunction; and (3) establish a cross-disciplinary framework to promote collaboration among medicine, physics, materials science, and engineering, thereby accelerating device development and clinical translation. Accordingly, this review covers the physicochemical properties of CAP, major classes of nanocarriers (e.g., liposomes, polymeric nanoparticles, magnetic nanocarriers), their synergistic mechanisms in modulating oxidative stress and BBB permeability, current therapeutic applications, existing challenges, and future perspectives.

Oxidative stress, defined as an imbalance between the generation of reactive oxygen species (ROS) and reactive nitrogen species (RNS) and endogenous antioxidant defenses, is a key pathological factor in the onset and progression of neurological diseases [4,5,6]. ROS, including superoxide anions (O_2_^•−^), hydrogen peroxide (H_2_O_2_), and hydroxyl radicals (^•^OH), as well as RNS such as nitric oxide (NO^•^) and peroxynitrite (ONOO^−^), when present in excess can damage intracellular lipids, proteins, and nucleic acids, leading to lipid peroxidation, protein misfolding, DNA damage, and subsequent cellular dysfunction [7,8]. Although disease manifestations differ, the contribution of oxidative stress is consistent across neurological disorders. In brain tumors, malignant cells exploit redox regulation and antioxidant defenses to resist chemotherapy and sustain uncontrolled proliferation [9]. During cerebral ischaemia–reperfusion injury, excessive ROS generation disrupts BBB integrity through degradation of tight junction proteins (e.g., occludin and claudin-5), induces neuronal apoptosis via mitochondrial dysfunction, activates inflammatory cascades through NF-κB signaling, and ultimately exacerbates infarct size and long-term neurological deficits [10]. In AD, oxidative stress accelerates β-amyloid (Aβ) aggregation, promotes tau hyperphosphorylation, and amplifies synaptic and neuronal loss [11]. Although antioxidant-based therapies have been developed, their clinical efficacy remains limited. A major obstacle is the BBB, a highly selective barrier formed by cerebral endothelial cells, astrocytic endfeet, and pericytes, which restricts approximately 98% of small-molecule drugs and nearly all macromolecules from entering the brain parenchyma [12]. Consequently, insufficient drug accumulation at lesion sites, reduced therapeutic efficacy, and increased systemic toxicity are common. Moreover, prolonged radiotherapy and chemotherapy often lead to drug resistance via efflux pump upregulation (e.g., P-glycoprotein), immune dysfunction, and secondary neurological impairments such as cognitive decline and peripheral neuropathy [13]. Therefore, achieving efficient BBB penetration alongside precise spatiotemporal control of oxidative stress remains a central challenge in contemporary neuroscience.

Nanomedicine has emerged as a promising strategy to address these limitations by exploiting advantages such as tunable particle size (1–100 nm), large surface area for high drug loading, controlled and stimuli-responsive release profiles, and versatile surface functionalization [14]. Diverse nanocarriers—including liposomes, polymeric nanoparticles (e.g., PLGA, chitosan), nanogels, micelles, metal nanoparticles, and quantum dots—have been extensively investigated for central nervous system (CNS) drug delivery [15]. These systems improve BBB penetration by optimizing pharmacokinetics; for instance, polyethylene glycol (PEG) modification prolongs circulation time by reducing clearance by the mononuclear phagocyte system (MPS). Furthermore, conjugation with brain-targeting ligands such as TAT peptide, rabies virus glycoprotein (RVG) peptide, or transferrin enables receptor-mediated transcytosis (RMT) across the BBB, thereby enhancing site-specific drug delivery to the CNS [16,17]. Despite these advances, several challenges persist. Many nanocarriers exhibit limited stability in circulation and are rapidly cleared by the MPS, resulting in reduced bioavailability [18]. Inadequate targeting specificity may lead to off-target accumulation in healthy organs, causing hepatotoxicity or nephrotoxicity [19]. In addition, therapeutic outcomes remain unsatisfactory for refractory brain diseases such as recurrent glioblastoma and advanced AD, due to pathological heterogeneity and complex microenvironments. Importantly, most nanomedical platforms are designed primarily for chemotherapy, limiting their integration with other physical therapeutic modalities that could provide synergistic benefits.

Plasma-based therapeutics have recently gained attention as a cross-disciplinary approach combining plasma physics, chemical engineering, and clinical medicine [20]. CAP, distinct from high-temperature industrial plasma, operates at atmospheric pressure and near-physiological temperatures (<40 °C), generating a complex mixture of biologically active components, including reactive oxygen/nitrogen species (RONS), low-intensity ultraviolet radiation, low-energy electrons, and weak electric fields [21]. This unique composition enables CAP to modulate cellular signaling pathways, regulate inflammatory responses, promote angiogenesis, and reversibly open the BBB without causing thermal damage [22]. Importantly, CAP can generate physiologically relevant levels of ROS and RNS, allowing fine regulation of oxidative stress. In brain tumors, CAP induces cancer cell apoptosis via ROS-mediated DNA and mitochondrial damage, inhibits angiogenesis, and enhances antitumor immunity [23]. In models of neurodegeneration and ischaemic injury, low-dose CAP modulates redox signaling, improves mitochondrial function, and exerts neuroprotective effects. Nevertheless, CAP therapy faces major delivery limitations: plasma devices cannot be directly applied to deep brain tissues, and the short-lived plasma-generated species are difficult to deliver systemically without rapid degradation.

The integration of plasma technology with nanomedicine offers a compelling solution to these challenges. Plasma can activate both aqueous systems and solid nanomaterials, imparting enhanced physicochemical and biological functions [24]. Plasma-activated water (PAW), for example, contains multiple RONS and exhibits antimicrobial activity with low cytotoxicity, while also acting as a signaling modulator for cellular processes [25,26]. Plasma treatment can further reactivate exhausted materials, extending their functional lifespan [27]. Conversely, nanomedicines provide efficient delivery, targeting, and controlled-release capabilities, facilitating BBB penetration and lesion-specific accumulation [28]. ROS-responsive nanocarriers and ligand-modified systems have demonstrated improved therapeutic precision in ischaemic and tumor models [29,30]. Collectively, plasma provides multifunctional activation and physical therapeutic effects, while nanomedicine ensures precise delivery and stimulus-responsive release, forming a synergistic platform for high-precision and minimally invasive brain disease therapy. Despite its promise, plasma–nanomedicine research remains at an early stage. Current studies are largely confined to glioma, AD, and ischaemic stroke, with limited exploration of depression and vascular cognitive impairment. Mechanistic understanding is incomplete, particularly regarding interactions between plasma-generated physical factors and nanomaterials, as well as their effects on glial and immune cells. Furthermore, standardized criteria for RONS dosage, plasma parameter optimization, and device calibration are lacking, hindering reproducibility and translation. These challenges are exacerbated by insufficient interdisciplinary communication and misaligned research priorities.

**Figure 1:**
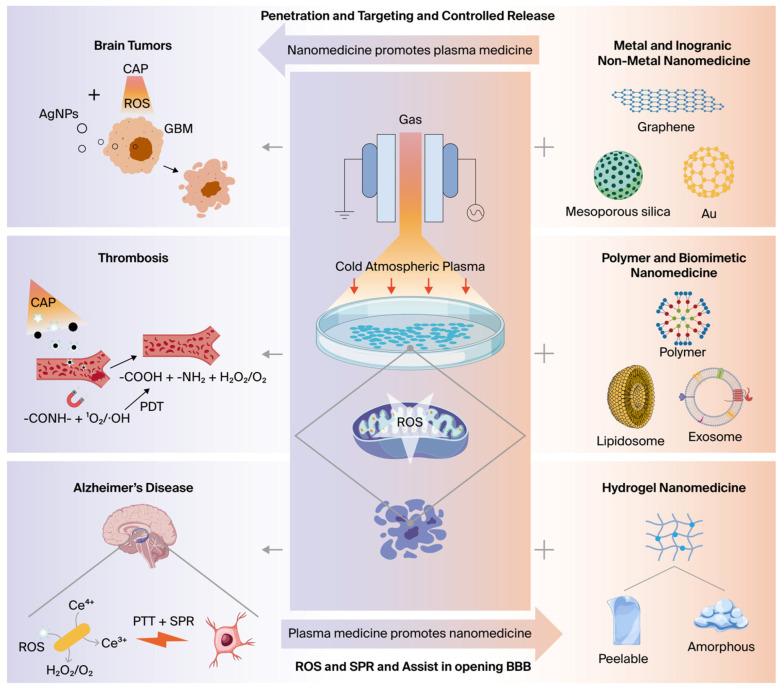
The schematic illustrates the synergistic application of CAP with various nanomedicines (including metal/inorganic, polymer/bionic, and hydrogel-based) as ROS-mediated therapeutic agents for the treatment of brain tumors, stroke, and degenerative neurological disorders. Created with BioGDP.com, accessed on 3 December 2025. [31].

Building on existing evidence, this review summarizes recent progress in plasma–nanomedicine synergistic therapy (Figure 1). By integrating physicochemical principles, nanocarrier design, and medical application—with particular attention to underexplored neuropsychiatric and cognitive disorders—this review aims to bridge disciplinary gaps, promote collaborative development of devices and protocols, and accelerate clinical translation. Ultimately, it seeks to advance precise brain disease therapies and establish new interdisciplinary pathways at the intersection of plasma science, nanotechnology, and medicine.

## 2. Overview of Plasma

### 2.1. Definitions and Classification

As the fourth state of matter that distinguishes itself from solids, liquids, and gases, plasma was originally systematically characterized by L. Tonks and I. Langmuir. Fundamentally, plasma is composed of partially or completely ionized conductive particle aggregates, including free electrons, charged ions, excited state and ground state atoms/molecules, and active free radicals [32]. Although plasma contains large numbers of charged particles, the total amount and spatial distribution of positive and negative charges are always in dynamic equilibrium, thus maintaining macroscopic electrical neutrality. This key feature makes it different from other charged particle systems. On a microscale, the interaction between plasma components gives it unique physicochemical properties. According to the thermal balance between electrons and ions, plasma can be categorized into two types: high-temperature plasma (about 10^9^ K) and low-temperature plasma (<10^3^ K). In high-temperature plasma, gases are nearly fully ionized, with electrons and heavy particles having very high thermal energy. In contrast, low-temperature plasma is in a partially ionized or non-ionized state, which can be further divided into thermal equilibrium type and non-thermal equilibrium type. In the thermal-equilibrium plasma, the temperature of electrons is similar to that of the ions and continues to be at a higher level. In contrast, in non-thermal plasma, the temperature of electrons can reach about 10^3^ K, while ions and atoms are cooled to about 300 K, close to room temperature. Because the macroscopic temperature of plasma is governed by heavy-particle temperature, such systems are termed cold plasma. When generated at atmospheric pressure, they are referred to as CAP. Owing to their ease of generation, near-physiological temperature, and safe interaction with biological tissues, non-thermal plasmas exhibit substantial research value and broad biomedical potential. CAP has consequently become an important technology in contemporary studies on plasma–nanomedicine synergistic therapy.

### 2.2. Biological Characteristics of CAP

Research shows CAP can generate over 50 chemical compounds and participate in more than 600 chemical reactions, with its chemical characteristics primarily determined by the composition of the feed gas [33]. By introducing various gases, including helium, argon, nitrogen, air, and oxygen, the types and concentrations of reactive substances can be precisely controlled [34]. ROS and RNS are the key components mediating the biological effects of plasma [35]. ROS mainly include ^•^OH, O_2_^•−^, singlet oxygen (^1^O_2_), ozone (O_3_) and H_2_O_2_, while RNS include NO^•^, nitrogen dioxide (NO_2_^•^) and nitrate (NO_3_^•^) [8]. Gaseous active species can further generate secondary active species (such as H_2_O_2_, OOH^−^, NO_2_^−^, ONOO^−^) upon contact with liquids (such as water, cell culture medium, tissue fluid). Based on their half-life differences, RONS are categorized into short-lived active particles and long-lived active particles (half-life > 1 h). The long-lived active particles include H_2_O_2_, NO_2_^−^, and NO_3_^−^, and the other active particles, such as ^•^OH, O_2_^•−^, ^1^O_2_, NO^•^, and ONOO^−^, are collectively called short-lived active particles [36]. These two types of species work synergistically to regulate the biological effects of CAP.

### 2.3. Applications of CAP Therapy

CAP, by leveraging the strong oxidizing effect of RONS, exhibits broad-spectrum antimicrobial activity and can efficiently inactivate bacteria, viruses, fungi, and their biofilms [37]. As early as 1968, Menashi [38] pioneered the use of CAP generated by pulsed radio frequency corona discharge to rapidly inactivate microorganisms in drinking water, achieving the elimination of 4 × 10^6^ microbial spores within 1 s. Subsequently, Shu et al. [39] demonstrated that an air-plasma hybrid system, through the synergistic O_3_/NO_x_ reaction, significantly enhances the oxidation environment in aqueous solutions, enabling complete eradication of Staphylococcus aureus within a short timeframe. In the field of oral medicine, CAP, which acts on tissues through gaseous active substances without causing mechanical or thermal damage, can eliminate bacteria within root canal biofilms within minutes and promote implant integration by regulating the expression of epithelial cell adhesion molecules [40]. CAP therapy has demonstrated remarkable potential in non-infectious disease treatments. In dermatological applications, Zheng et al. successfully treated reverse psoriasis using plasma jet technology [41], while Zhai et al. achieved effective intervention for vitiligo and psoriasis by combining atmospheric pressure plasma nanoparticles (APNP) with hydrogel [42]. Within the domain of wound healing, Choi et al. [43] found that cold plasma can induce nuclear translocation of β-catenin while concurrently elevating the expression of c-MYC and Cyclin D1 genes, thereby enhancing the proliferation and migration of dermal cells and inhibiting bacterial infection at the same time. In oncology, Qi et al. demonstrated that combining CAP-activated saline with peritoneal hyperthermia significantly reduced tumor mass and prolonged survival in peritoneal cancer mice [44]. Building upon its established applications in infectious diseases, dermatological disorders, wound healing, and cancer therapy, plasma technology has been increasingly applied to autoimmune disease intervention, neurodegenerative disease adjunctive treatment, and precision ophthalmic therapy. While maintaining core mechanisms centered on RONS (Regulatory On–Off Switching) precision control, targeted cellular signaling modulation, and immune microenvironment remodeling, the technology exhibits disease-specific optimization in both therapeutic targets and technical approaches. Since Laroussi [45]. First reported the sterilization effect of APNP on Escherichia coli in 1996, the research on plasma–nanomedicine synergistic system in the biomedical field has been deepened, and it has shown a wide range of application prospects in sterilization, disinfection, and disease treatment.

### 2.4. Neuroprotective Mechanism of Atmospheric Pressure Plasma Jet (APPJ)

**Figure 2:**
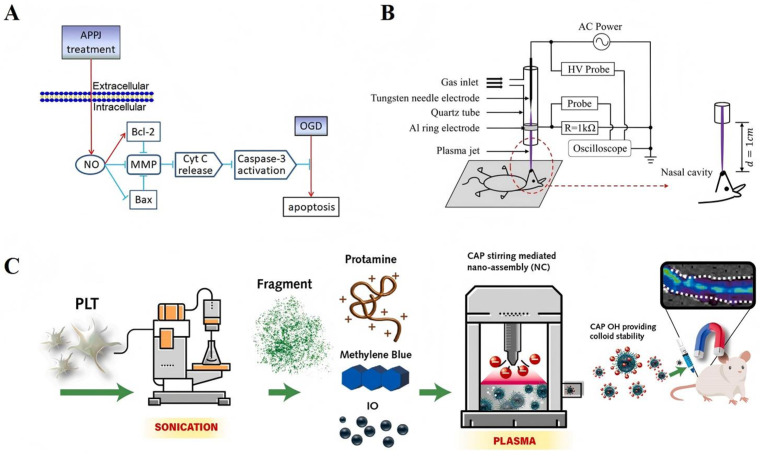
(**A**). APPJ attenuates OGD-induced apoptosis, thereby protecting neurons [46]. Red arrows represent promotion, and blue arrows represent inhibition. Copyright © 2025, Wiley-VCH GmbH. (**B**). The plasma jet was injected directly into the nasal cavity of rats for inhalation [48,49]. Copyright © 2025, Frontiers in Neuroscience. (**C**). This nanomaterial was created by utilizing CAP to irradiate MB, PLTV, and IO with varying concentrations of protamine Pro, then mixing them all together. In order to achieve distant photolysis of thrombus, the study used NIR irradiation to induce PDT and PTT, and a static magnet to direct PLTV-IO-MB PENPs into deep blood clots [51]. Copyright © 2025, ELSEVIER.

APPJ is a kind of non-equilibrium plasma jet generated under normal pressure. It can be produced in an open space without the need for a vacuum environment, and it is widely applied in medical and industrial fields. In 2020, an oxygen–glucose deprivation (OGD) model simulating ischemia—reperfusion injury was used to assess the neuroprotective effects of APPJ (Figure 2A) [46]. A quick 24 s treatment increased nerve cell survival by 1.5 times after optimizing the APPJ device parameters. Mechanism-level verification shows that APPJ influences the mitochondrial apoptotic pathway by raising NO levels: it inhibits both the activation of caspase-3 and the release of cytochrome from mitochondria while also upregulating anti-apoptotic Bcl-2 protein expression, and downregulating pro-apoptotic Bax protein levels. In the end, this lessens the death of neurons brought on by OGD. In addition, cell survival in the APPJ group decreases by 50% upon the introduction of a NO scavenger, indicating that NO is the essential mediator by which APPJ exerts neuroprotective effects. Additionally, it was discovered in 2022 that APPJ could significantly increase the intracellular concentration of cyclic guanosine monophosphate (cGMP) to three times that of the expression of PKG in the control group increased by a factor of two. The neuroprotective effect of APPJ was completely eliminated when the cGMP route was stopped using a particular inhibitor, indicating that the NO/cGMP/PKG signaling pathway is the primary molecular mechanism by which APPJ offers its neuroprotective advantages [47]. Ye et al. [48,49] used helium as the working gas; they created a needle-ring structured dielectric barrier discharge (DBD) type APPJ and administered intermittent inhalation treatment to rats suffering from middle cerebral artery occlusion (MCAO) (Figure 2B). Experimental findings revealed that the APPJ treatment group’s local cerebral blood flow was 1.7 times greater than that of the MCAO model group, the area of cerebral infarction was only 42% smaller than that of the MCAO group, and there were significantly fewer apoptotic cells in the brain tissue. The above therapeutic effects are closely associated with the formation of NO caused by plasma, according to the mechanism analysis. The neuroprotective impact of APPJ in living things is further supported by NO’s ability to widen cerebral blood vessels, improve local blood flow, and concurrently inhibit neuronal death.

### 2.5. Thrombus Treatment

Worldwide, stroke constitutes the leading etiology of mortality and disability. The loss of nerve cells and the rupture of the blood–brain barrier brought on by cerebral ischemia–reperfusion constitute its primary pathogenic harm [50]. The RONS generated by APPJ have shown significant neuroprotective effects, while the preparation of plasma-assisted functional nanomedicines has significantly enhanced thrombolytic performance, jointly providing an innovative therapeutic strategy. In order to restore blood flow during stroke treatment, the thrombus must be removed. The Chuang group created functionalized nanomedicines that increase the efficacy of thrombolysis using plasma-assisted techniques. Using a CAP-assisted stirring method, they produced composite nanoclusters made of methylene blue (MB), curcumin (Nu/Fu), and iron oxide (IO) (MB-Nu/Fu-IO). The CAP prevents these nanoclusters from aggregating within living things and enhances their colloidal stability. This nanocluster exhibits relatively low toxicity to normal vascular endothelial cells and achieves a thrombus clearance rate of approximately 80% by the combined action of near-infrared photothermal effects, photodynamic effects, and magnetic targeting [51]. Additionally, the team created CAP-assisted self-assembled PLGA-TV peptide-IO-MB nanopropellers (PLTV-IO-MB) (Figure 2C), where the TV peptide selectively targets platelets on the thrombus surface to increase the efficiency of nanomedicine accumulation at the thrombus site. This nanofluidic thruster addressed the clinical problem that traditional thrombolytic medications (such as alteplase) frequently result in bleeding issues by showing an 80% efficacy in dissolving clots in the mouse tail vein thrombosis model without any discernible danger of bleeding [52]. Nevertheless, the adaptation verification for various thrombi (such as cerebral thrombi) is not mentioned, and this treatment necessitates a suitable NIR light source. Its clinical application value can be further increased in the future by creating a smaller NIR magnetic control system, simplifying the CAP device, and covering various thrombi and populations.

Plasma–nanomedicine has shown two benefits in the treatment of stroke: on the one hand, by controlling RONS (particularly NO) and its downstream NO/cGMP/PKG signaling pathway, it has neuroprotective effects at the cellular and animal levels; on the other hand, it can be used as a tool to maximize the structural stability and targeting of nanomedicine and enhance thrombolytic effects. This offers a fresh approach to the precise and multimodal treatment of stroke.

## 3. Types of Nanomedicines in Plasma–Nanomedicine

Plasma–nanomedicine is a cutting-edge treatment system that organically integrates the active factors generated by plasma with nanomaterials to build an efficient drug delivery and precision treatment platform. In contrast, plasma–nanomedicines, by coordinating the structural tunability of nanomedicines with the plasma-induced generation of active substances, photothermal effects, and energy conversion characteristics, can not only significantly enhance the tissue permeability and cellular uptake efficiency of drugs, but also amplify redox reactions, photothermal therapeutic effects, and catalytic activity. This enables a multi-dimensional collaborative treatment model of “energy-driven functional response”. Based on the differences in composition, plasma–nanomedicines are mainly classified into types as metal and inorganic non-metal nanomedicines, polymer nanomedicines, biomimetic nanomedicines, and hydrogel nanomedicines. With their unique physicochemical properties, various materials have demonstrated advantages over traditional drug systems in key links such as drug delivery, oxidative stress regulation, and transmembrane transport, providing a brand-new technical path to break through the limitations of classic treatment strategies.

### 3.1. Metal and Inorganic Non-Metal Nanomedicines

**Figure 3:**
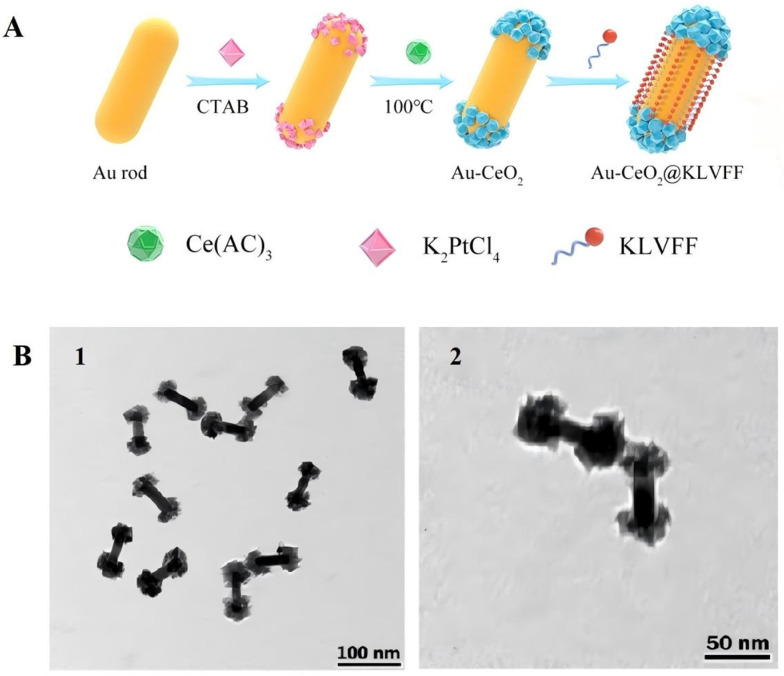
(**A**). CuO_2_ is selectively coated on Au NRs to create Au-CeO_2_ nanostructures that resemble dumbbells. (**B**). The TEM image shows that the synthesized Au-CeO_2_ is uniformly dispersed in the form of dumbbells, with a length of approximately 80 nm [58]. The magnification of (**B2**) is twice that of (**B1**). Copyright © 2025, ACS Publications.

Metal nanomedicines such as gold and silver, owing to their excellent local surface plasmon resonance (LSPR) properties, can effectively amplify optical signals, enhance functional reactivity, optimize energy utilization efficiency, and have broad applicability in therapeutic applications for brain diseases, targeted drug delivery, and biosensing fields [53,54,55]. In addition, iron nanoparticles improve the permeability and brain accumulation of the blood–brain barrier by producing local heat therapy, which is used for the diagnosis and treatment of the CNS [56]. Furthermore, cerium nanoparticles go through a complex “in vivo processing” process, which is closely related to their final biological effects (such as antioxidation or oxidation) [57]. Gao et al. [58] prepared uniform-sized gold nanorods (Au NRs) by seal-mediated growth method and adopted a spatial separation structure design strategy to selectively deposit cerium oxide nanoparticles (CeO_2_ NPs) at both ends of Au NRs, successfully constructing dumbbell-shaped Au-CeO_2_ composite nanomaterials. When subjected to near-infrared (NIR) light exposure, the thermal electrons generated by Au NRs through the plasma photothermal effect are injected into the CeO_2_ conduction band, accelerating the valence state conversion of Ce^4+^ to Ce^3+^, and significantly enhancing the photocatalytic activity and redox performance of the material. Empirical findings revealed that the combined treatment group of Au-CeO_2_ + NIR had the best decomposition ability for H_2_O_2_, which could mitigate the intracellular ROS level of PC12 cells to a level close to normal, and its fluorescence intensity was only about one-third of that of the positive control group. It effectively alleviated the oxidative stress damage caused by the aggregation of Aβ in AD. Meanwhile, this combined system has demonstrated significant advantages in enhancing cell survival rate and inhibiting Aβ-induced apoptosis, providing a solid experimental support for its application in the treatment of AD (Figure 3A,B). Despite the fact that K-CAC nanocomposites have produced multimodal synergistic therapy, clinical translation is challenging due to their complicated production process, uncertain metabolism and safety in vivo, and inadequate NIR irradiation penetration in deep brain areas. In addition, it is worth noting that the self-redox activity of cerium dioxide nanoparticles can show oxidation-promoting or antioxidation bidirectional regulation in different microenvironments, which deeply affects the neuroimmune homeostasis [59].

**Figure 4:**
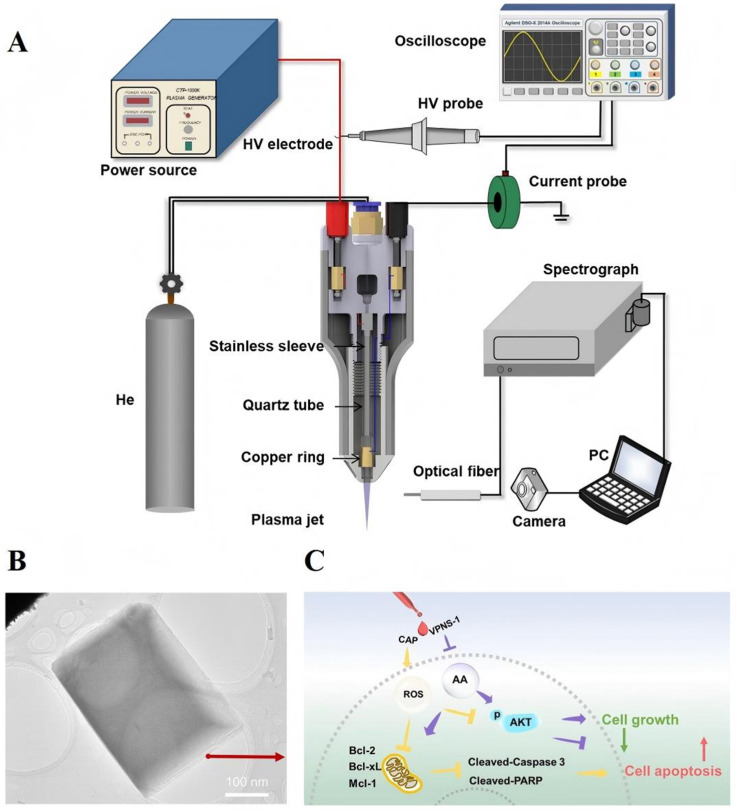
(**A**). Schematic diagram of the CAP emission device. (**B**). Transmission Electron Microscopy (TEM) image of VPNS. (**C**). The VPNS-1 and CAP synergistic impact in cancer cells is depicted in the schematic diagram: cell growth can be inhibited, and apoptosis can be induced when VPNS-1 and CAP are combined to suppress the expression of anti-apoptotic proteins of the Bcl-2 family (Bcl-2, Bcl-xL, and Mcl-1). The yellow and purple T-shaped arrows represent inhibition, while the yellow and purple arrow represents process [61]. Copyright © 2025, ELSEVIER.

Inorganic nanomedicines possess unique physical and chemical properties (such as catalytic activity, optical performance, thermal performance, electrical performance, and magnetic performance, etc.), which can achieve multiple functions such as drug delivery, disease diagnosis, bioimaging, and targeted therapy, and have broad application prospects in the biomedical field [60]. Xu’s team [61] designed a novel purple phosphorus nanosheet (VPNS-1) (Figure 4B), which, through its synergistic effect with CAP, constructed an efficient tumor treatment system (Figure 4A). The ROS/RNS produced by CAP directly act on the lipid bilayer of the cancer cell membrane, triggering lipid peroxidation reactions, disrupting the integrity of the membrane structure, resulting in a marked elevation of cellular membrane permeability, thereby promoting the intracellular efficiency of VPNS-1 and reducing the effective concentration and toxic side effects of nanomedicines. Meanwhile, high concentrations of ROS/RNS can directly induce oxidative stress damage in cancer cells (Figure 4C). Experimental data show that in the combined CAP-VPNS-1 treatment cohort, tumor volume inhibition achieved a 61.4% reduction relative to the control arm. This synergistic effect is achieved through a dual pathway of “enhanced membrane permeability” and “biochemical regulation (inhibition of metabolic pathways + induction of oxidative stress)”, effectively enhancing the anti-tumor effect and reducing therapeutic toxicity. However, this plasma device can only treat surface or shallow cancers because the plume it produces is only approximately 3 cm long. As a result, it can not adequately penetrate deep tissue malignancies. Furthermore, it is expensive to use pure helium as the working gas in large-scale, long-term research or therapeutic settings. In order to cut expenses, it may be investigated in the future to employ a combination of helium and a tiny amount of oxygen or nitrogen without lowering activity.

Therefore, the combined application of metal nanomedicines and plasma has the dual advantages of physical energy conversion and chemical reaction regulation, but it still has problems such as complex structural design, strict preparation process requirements, insufficient stability of surface modification, and the in vivo metabolic mechanism and long-term safety being not yet fully clarified. Inorganic non-metallic nanomedicines have become key materials in the nanomedicine domain and biosensing due to their excellent optical, electrical, and chemical tunability. However, their potential biological toxicity, poor in vitro and in vivo stability, and other limitations still need to be addressed. Overall, through the synergistic integration of external plasma stimulation and nanomedicines, more efficient disease treatment effects can be achieved.

### 3.2. Polymeric and Biomimetic Nanomedicines

**Figure 5:**
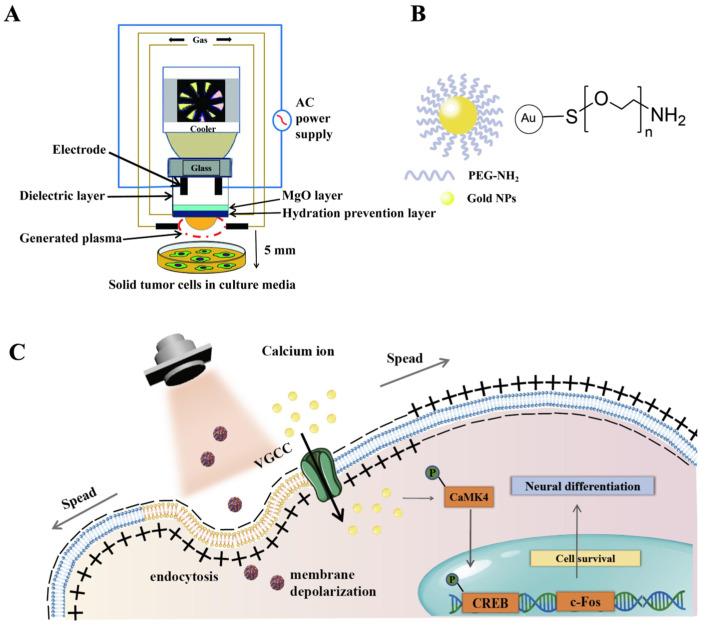
(**A**). Schematic of plasma treatment experimental apparatus. (**B**). The structure of polyethylene glycol coated with gold nanoparticles. (**C**). Schematic diagram of the mechanism of accelerating NSC directional differentiation through CS nanoparticles and NIR-II laser irradiation.

Polymeric nanomedicines encompass two major categories: synthetic polymeric nanomedicines and natural polymeric nanomedicines. Among synthetic polymeric nanomedicines, PLGA, PEG, polyurethane, polycaprolactone (PCL), and polyhydroxyacetic acid are typical representatives [62]. These nanomedicines exhibit excellent biocompatibility, controlled drug release properties, and passive tumor accumulation (strengthened permeability and retention effect, EPR effect) [63]. They can respond to internal stimuli (such as glutathione, ROS, and overexpressed enzymes) and external stimuli (such as light, sound, and magnetism), showing unique advantages in disease treatment [64,65,66]. Kaushik et al. treated glioblastoma cells with 30 nm 30 nm PEG-coated gold nanoparticles (GNPs) combined with CAP. The results show that the intracellular ROS level is increased by 1.5–2 times, and at the same time, it effectively inhibits the PI_3_K/AKT signaling pathway and induces more than 33% of tumor cell apoptosis. In the mouse xenotransplant tumor model, compared with the control group, the synergistic strategy reduced the volume and weight of the tumor by 50%, showing significant anti-tumor activity (Figure 5A,B). In addition, this plasma apparatus is structurally tailored to meet the needs of biomedical research; it has no heat damage, is easy to use, produces active species effectively, and works with standard cell culture supplies. Its use is restricted to small-scale farming operations, though. Additional tuning is still required if it is to be developed further for clinical usage. Li et al. [67] used bovine serum albumin (BSA) to functionalize copper selenide nanoparticles (Cu_2−x_Se NPs, CS NPs), and further improved their biocompatibility through PEG modification. CS NPs showed LSPR absorption characteristics in the second near-infrared window (NIR-II). When 1064 nm lasers are activated together, they can efficiently induce the directional differentiation of neural stem cells (NSCs) into mature dopaminergic neurons, providing a new treatment strategy for PD (Figure 5C).

**Figure 6:**
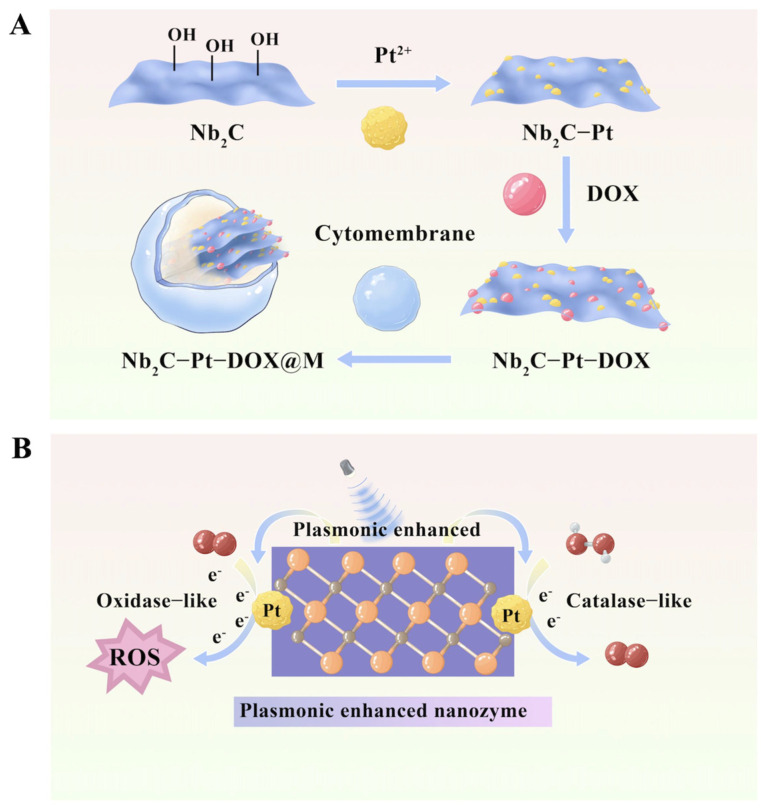
(**A**). Preparation of bionic plasma assembly NPD@M. (**B**). The catalytic process of plasma-enhanced nanoenzymes. Deep tumor cells were killed by the many thermal electron oscillations produced by NIR-II laser excitation of Nb2C, which increased the nanoenzyme’s catalytic activity.

Bionic nanomedicines are new nanomaterials designed by simulating natural biological structures (such as cell membranes) in nature. The material is composed of a functional nanomedicine core and a bioactive cell membrane coating. It has the core characteristics of immune escape, targeted delivery, and long-term circulation, and is an ideal drug delivery carrier [68]. Gao et al. designed a bionic plasma assembly (NPD@M) based on MXene [69]. By preparing ultrathin Nb_2_C plasma materials and platinum (Pt) nanoenzymes, the researchers loaded them on the surface of Doxin (DOX) and encapsulated them in the tumor cell membrane, successfully building the NPD@M nanomedicine system (Figure 6A). As a plasma material, MXene (Nb_2_C) exhibits thermoelectron oscillation under NIR-II laser irradiation, which significantly enhances the hydrogen peroxide-like and oxidase-like activity of platinum nanoenzymes (Figure 6B). The plasma enhancement effect significantly increases the generation of oxygen (O_2_) and ROS, which effectively kill tumor cells through ROS-mediated oxidative stress, and alleviate tumor microenvironmental hypoxia. The experimental results showed that relative to the control group, the survival rate of tumor cells in the NPD@M + laser (L) treatment group was significantly reduced by 38.67%, which fully verified the effectiveness of plasma-enhancing catalytic effect in tumor treatment. However, complicated procedures like high-temperature etching and ultrasonic stripping are needed to create this nanomaterial. Furthermore, Pt nanoenzymes are made of precious metal, which raises material costs and makes it challenging to satisfy the financial needs for widespread clinical use. To increase the possibility of clinical transformation in the future, the preparation procedure must be made simpler, and the drug release specificity must be optimized.

In summary, although polymer nanomedicines have excellent biocompatibility, their stability is susceptible to external environmental factors, which often leads to structural degradation or uneven drug release. Bionic nanomedicines have become key carriers for drug delivery and disease treatment because of their structure highly similar to natural cell membranes, excellent biocompatibility, and inherent targeting ability. However, there are still significant limitations in the fields of biosafety assessment and precise functional regulation, which cannot be ignored.

### 3.3. Hydrogel Nanomedicines

**Figure 7:**
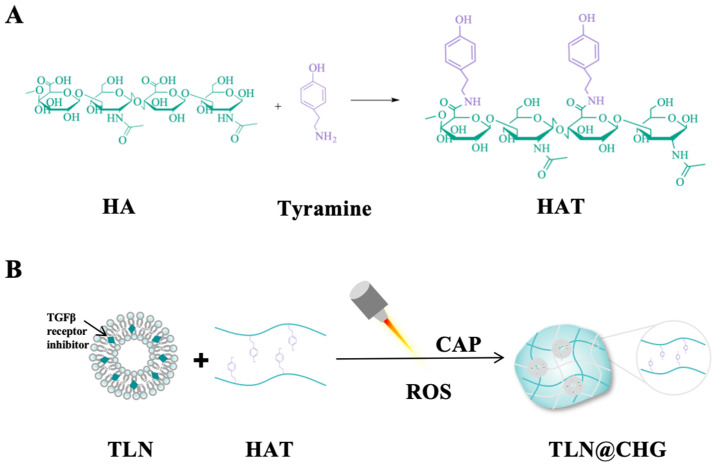
(**A**). The chemical structure of HAT and its CAP-induced cross-linking mechanism are depicted in the picture. (**B**). The CAP-induced hydrogel matrix encapsulates TLN (TLN@CHG), which is produced when the HAT-loaded nanoparticles undergo the phenolic polymerization reaction of HAT.

Hydrogel nanomedicine is a three-dimensional hydrophilic networked system formed through physical or chemical crosslinking, which can be divided into single polymer networks and composite nanomedicine hybridisation systems [70]. Among them, the composite nanomedicine hybridisation system has a broader application prospect because of its drug-carrying ability, controlled release characteristics, and environmental responsiveness. The Byun group [71] designed lipid nanomedicine (TLN, particle size about 112 nm), loaded with transformation growth factor-β receptor kinase inhibitor (TRKI), and constructed a CAP-responsive in situ hydrogel (TLN@CHG) loaded with TLN (Figure 7). The ROS produced by CAP not only triggers the phenolic polymerization reaction of hyaluronic acid-tyramine coupling (HAT) to achieve in situ gelation, but also elicits immunogenic cell death in tumor cells. This changes the hydrogel from a simple “drug carrier” to an “in situ vaccine”. In the mouse model, the system achieved the complete regression of the primary tumor, induced 100% long-term survival rate, and had a systemic immune memory effect. However, specific tools and procedures are crucial to this research. Certain CAP devices are needed. Furthermore, this nanomaterial has not been studied on tumors with lesser immunogenicity (such as glioblastoma, etc.), and its effectiveness has only been confirmed in animal models of breast and colorectal cancer. As a result, there are many opportunities to broaden the scope of research. In summary, the hydrogel-encapsulated nanodrug system has become an innovative treatment platform with great potential in the domain of biomedicine; the integration of plasma technology offers comprehensive advantages of targeted, controllable, and precise release while protecting drug activity.

### 3.4. Redox Mechanism of Plasma–Nanomedical Synergy

CAP and PAW produce a variety of mixtures of RONS, including short-lived oxygen (^•^OH, O_2_^•−^, ^1^O_2_ and ONOO^−^) and long-lived oxygen (H_2_O_2_, NO_2_^−^ and NO_3_^−^) [35]. Abundance and lifespan of these species depend on the composition, humidity, discharge mode, and exposure time of the raw gas [33,36]. Quantitative analysis of PAW shows that H_2_O_2_ usually accumulates in the range of 10–300 μM, while NO_2_^−^ and NO_3_^−^ often reach 20–80 μM after a few minutes of plasma exposure [26,27]. Short-lived species dominate the initial oxidation outbreak, while long-lived species maintain downstream redox signals, so that the treatment results depend on their joint dynamics [26].

#### 3.4.1. Hormone Redox Regulation of the Nervous System

In nerve tissue, RONS produced by CAP exhibit distinct activation patterns: low-to-moderate levels activate adaptive antioxidant responses via Nrf2, upregulating enzymes such as NQO1, SOD, and GPX, thereby restoring redox homeostasis under ischemic or neurodegenerative stress [72]. NO produced when exposed to CAP also activates the NO/cGMP/PKG pathway, improves microcirculation, and promotes neuronal survival [47]. For example, APPJ triples the expression of cGMP in cells and doubles the expression of PKG, and the inhibition of the cGMP pathway eliminates the neuroprotective effect. On the contrary, excessive ONOO^−^ or •OH can lead to lipid peroxidation, mitochondrial failure, ferroptosis, and DNA damage [72]. Therefore, the treatment safety window of CAP is defined by RONS concentration, exposure time, and tissue penetration behavior [47].

#### 3.4.2. Nanomedicine Interacts with CAP by Controlling the Spatial and Temporal Distribution of RON and Directly Changing the Redox Behavior

In redox-active nanoenzymes (for example, CeO_2_, MnO_2_), CeO_2_ NPs show a redox cycle between Ce^3+^/Ce^4+^ states, which can effectively scavenge O_2_• and •OH, buffer oxidative outbreaks caused by CAP, and prevent lateral branch neurotoxicity [58]. Under near-infrared excitation, the Au-CeO_2_ composite further accelerated the transformation of Ce^4+^ → Ce^3+^ and effectively reduced intracellular ROS in the neuron model [58]. Furthermore, organic frameworks (MOF) and polymer carriers show unique advantages in this field. MOF and polymer matrix preserve the long-life particle beams produced by plasma, prolonging their half-life and enhancing the deep diffusion of tissues [26,27,73]. Some metal-containing systems participate in Fenton/Fenton-like reactions, amplifying the therapeutic oxidative stress of tumors, but strict dose control is required in neural settings. Nanomedicine that releases NO or nanomedicine with NO stabilization also shows good application prospects. CAP-generated NO can be wrapped in hydrogel or liposomes to produce continuous vasodilation, anti-inflammatory effects, reduce the activation of small glial cells, and supplement the endogenous nerve protection NO/cGMP pathway [46,47,48,49].

In summary, metal-based, inorganic non-metals, polymers, bionic nanomedicine, and hydrogel nanomedical systems provide critical tools for the diagnosis and treatment of a variety of diseases, especially in the field of brain diseases. Its regulable size and surface characteristics enable receptor-mediated endocytosis to cross the BBB so as to accomplish site-specific drug delivery and improve bioavailability. However, there are still some shortcomings. For example, the majority of research concentrates on conventional nanoparticles like gold and silver, but the use of novel carriers like composite oxides is still very restricted. Furthermore, there is currently a dearth of research on the combination of CAP and nanoparticle therapy in other brain diseases, such as neurodegenerative disorders and mental disorders, making it impossible to perform a thorough and methodical evidence-based evaluation. Given this, future studies should concentrate on the mechanism analysis of this combination therapy, expedite the creation of multifunctional nanocarrier materials and high-performance medical CAP devices, and increase their use in the treatment of brain disorders (Table 1).

## 4. Plasma–Nanomedicine Therapy for Brain Diseases

Brain diseases are characterized by high rates of disability, recurrence, and treatment difficulty, making them a major public health issue that threatens human life, health, and family well-being [80]. There are significant restrictions on the available treatments for brain illnesses. In medical treatment, the physical and biological defenses of the BBB hinder most small-molecule and large-molecule medications from passing through and reaching the target area at therapeutic levels [81]. Significant toxicity and adverse consequences might also result from non-specific dispersion throughout the body [82]. Although local lesions can be immediately targeted by surgery, invasive lesions (such as gliomas) are difficult to completely eradicate and may permanently harm the surrounding healthy brain tissue, leading to neurological deficits [83].

The synergy of CAP and nanomedicine lies in precisely regulating the redox balance in the microenvironment of brain diseases. Its molecular mechanism can be divided into three dimensions: the bidirectional regulatory pathway mediated by RONS, the modulation of the antioxidant enzyme system, and the targeted blocking of disease-specific oxidative stress circuits. In addition, there are significant variations in the regulatory properties of various nanomaterial systems. The main mediators that control oxidative stress are active oxygen/nitrogen species (lon) produced by CAPs such as NO, ^•^OH, O_2_^•−^, and H_2_O_2_. Nanomedicines use targeted distribution and sustained release of the microenvironment to precisely regulate RONS in both space and time. According to research, low-dose ion can increase the nuclear translocation efficiency of nuclear factor erythroid 2-related factor 2 (Nrf2) by activating the cGMP/PKG signal axis, and low-dose PAW is downstream anti-ferop. It has been shown that Tosis can be activated. The pathway by promoting the nuclear translocation of Nrf2 [84]. This keeps cells’ redox equilibrium moderate and promotes oxidative homeostasis (eustress). In contrast, high doses of PAW significantly inhibit NrF2 activity, reduce the expression level of GPX4, lead to the collapse of the intracellular antioxidant defense system, and cause an oxidative stress response. In conclusion, the combination of plasma medicine and nanomedicine can provide a novel mechanism for the therapy of brain diseases and enhance therapeutic efficacy and specificity.

### 4.1. Brain Tumor Treatment

Brain tumors, especially glioblastoma multiforme (GBM), the most common malignant tumor in the CNS, are characterized by strong treatment resistance, high invasiveness, and difficulty passing through the BBB [85]. Existing standard clinical treatment approaches, including postoperative radiation therapy and temozolomide (TMZ) chemotherapy, followed by maximum safe surgical removal, have limited effectiveness; the median survival of patients is only 12 to 15 months, and the prognosis is very poor [86]. By utilizing the combined impact of many pathways, plasma–nanomedicine offers a novel treatment strategy for GBM.

For instance, Keidar’s group has thoroughly examined plasma’s anti-tumor properties, laying theoretical groundwork for upcoming combination treatments. Plasma’s targeted benefit in anti-tumor therapy was first demonstrated in 2011, when it was confirmed that it could specifically kill cancer cells by initiating the apoptotic pathway while showing comparatively low toxicity to normal brain cells [87]. Then, ROS produced by CAP can cause tumor cell death, according to research on microenvironmental regulation conducted in 2019. Its anti-tumor efficacy can also be increased by modifying the tumor microenvironment, such as by preventing the creation of tumor blood vessels and reducing the infiltration of immunosuppressive cells [88]. A synergistic effect between the two was confirmed in 2020 when in vitro collaborative experiments using a GBM cell model showed that the combined treatment of CAP and TMZ significantly heightened DNA damage, resulted in cell cycle arrest at the G2/M phase, and decreased tumor cell viability by 20% when relative to TMZ monotherapy [89]. In vivo effectiveness tests conducted in 2021 revealed that the CAP/TMZ combination treatment resulted in a 4.4-fold decrease in tumor volume in a mouse cerebral GBM model. However, the study also showed that the active particles produced by CAP were unable to enter the skull directly, which limited its direct application in the treatment of brain malignancies [90].

**Figure 8:**
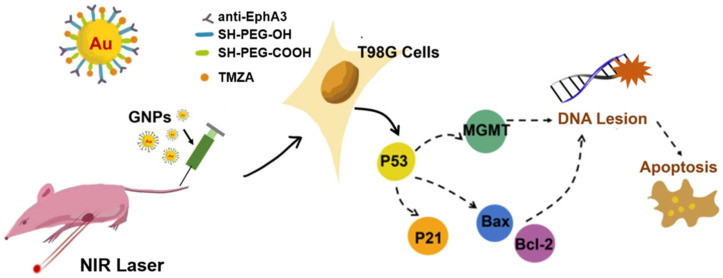
Schematic illustration of anti-EphA3-TMZ@GNPs-induced apoptosis in tumor cells [79]. Copyright © 2025, ACS Publications.

Researchers have combined plasma with functionalized nanomedicines to overcome the problem that plasma can not pass through the skull. This allows for targeted medication delivery and increases the plasma’s action through nanocarriers. Yu et al. [79] developed gold nanoparticles averaging approximately 45 nm in diameter that were coupled with TMZ and altered with anti-EphA3 antibodies (anti-EphA3-TMZ@GNPs). Through intranasal delivery, this technique penetrates the BBB and targets GBM cells by attaching the EphA3 antibody to the highly expressed EphA3 receptor on their surface. Furthermore, gold nanoparticles’ plasmonic photothermal action amplifies TMZ-induced DNA alkylation (Figure 8). According to experimental results, the group treated with anti-EphA3-TMZ@GNPs in conjunction with near-infrared laser achieves an inhibition rate of 88.32%, greatly increasing the therapeutic effectiveness, while the tumor inhibition rate with TMZ treatment alone is only 11.33%.

**Figure 9:**
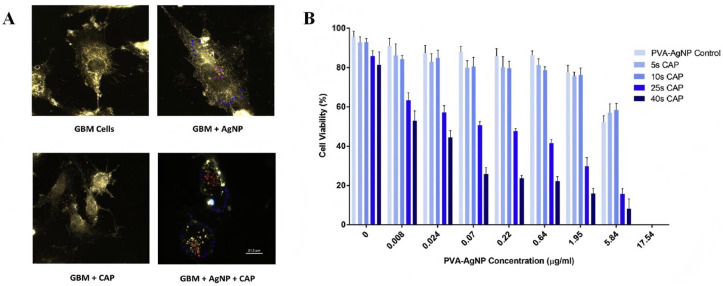
(**A**). Schematic of AgNP oxidation and dissolution induced by CAP. Representative spectral response of GBM cells (blue), background (black), PVA-AgNP (magenta) in cells and the combined treatment of AgNP-CAP (red). (**B**). Effects of the combination of AgNP and CAP therapy on glioblastoma cells [77]. Copyright © 2025, ELSEVIER.

An advanced study on how CAP enhances the cellular uptake of metal nanomedicines has been conducted by He’s team. In 2018, by promoting ATP-dependent endocytosis, it was discovered that CAP significantly increases the absorption of AuNPs in GBM cells, leading to a 25-fold increase in AuNP cytotoxicity compared to treatment with AuNPs alone [75]. It was further demonstrated in 2020 that clathrin-mediated endocytosis (CME) is the principal mechanism by which CAP increases the uptake of AuNPs, providing molecular knowledge for regulating the effectiveness of nanomedicine internalization [91]. In the same year, Manaloto et al. [77] discovered that CAP might change the physicochemical characteristics of polyvinyl alcohol (PVA)-coated silver nanoparticles (AgNPs). By reducing the initial particle diameter from 10 nm to 5 nm and the zeta potential to −26.8 mV, moderate drug aggregation was caused. This structural change tripled the absorption of AgNPs by GBM cells, increased the discharge of Ag+ by 2.5 times, and ultimately doubled the mortality rate of GBM cells, providing a new approach for plasma-based regulation of nanomedical activity (Figure 9A,B).

In conclusion, improved targeting, physicochemical manipulation, and biochemical amplification provide synergistic therapeutic benefits when plasma and nanomedicine are combined. This combination strategy offers a viable path for future clinical translation in the treatment of GBM, whether through plasma-induced reactive species or plasma-driven enhancement of nanomedicine performance.

### 4.2. Neurodegenerative Diseases Treatment

Neurodegenerative diseases (NDDs) are a set of long-lasting conditions marked by the gradual degeneration of neurons, such as amyotrophic lateral sclerosis (ALS), PD, AD, and more [92]. According to WHO estimates from 2021, around 3.4 billion people worldwide—or 43.1% of the total population—have neurological illnesses [1]. AD is the most frequent of these conditions, followed by PD [93]. PD is marked by the loss and degeneration of dopaminergic neurons in the substantia nigra pars compacta [94], whereas AD pathogenesis focuses on extracellular Aβ plaque buildup and intracellular neurofibrillary tangles containing hyperphosphorylated tau [95]. Current treatments reduce symptoms but do not stop or alter the course of the disease. By focusing on protein aggregation and encouraging brain regeneration, plasma–nanomedicine presents new therapeutic possibilities.

**Figure 10:**
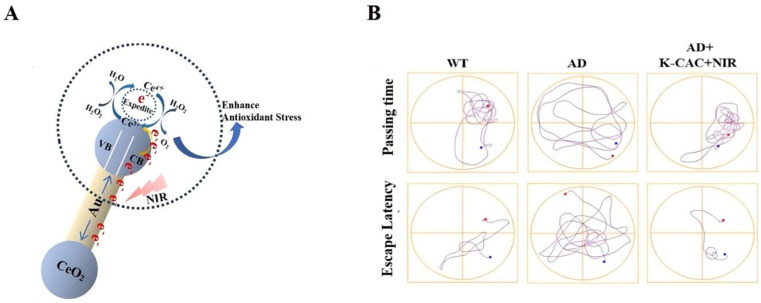
(**A**). Mechanism of cerium oxide catalysis enhanced by NIR-induced plasma hot electron injection. (**B**). Morris water maze experiment [58]. Copyright © 2025, ACS Publications.

In response to the pathogenic feature of Aβ aggregation in AD, Gao et al. [58] created Au-CeO_2_@KLVFF nanocomposites. With CeO_2_ NPs placed on the surface and modified with the Aβ-targeting peptide KLVFF (which may specifically attach to Aβ fibrils), Au NRs form the core of this structure. Among them, the BBB tight connections can be locally opened by gold nanorods’ plasmonic photothermal action, which increases the BBB’s permeability by roughly four times and makes it easier for the nanocomplexes to reach brain tissue. While the redox activity of CeO_2_ nanoparticles is significantly increased under near-infrared light, enabling them to efficiently break down Aβ fibrils and reduce the formation of senile plaques, the KLVFF peptide guides the nanocomplexes to precisely target and bind to Aβ fibrils (Figure 10A). This nanocomposite treatment raised the hippocampal neuron survival rate from 67.3% to 90.4% in the APP/PS1 double transgenic AD mouse model. The Morris water maze experiment demonstrated that cognitive function in mice was greatly improved, giving a new technique for the pathological intervention of AD (Figure 10B).

Gao et al. [67] copper selenide (Cu_2−x_Se) nanoparticles (CS NPs) in PD therapy to encourage dopaminergic neuron regeneration. Strong NIR-II (1064 nm) LSPR absorption by CS NPs produces localized heat and low-level ROS, which trigger neural stem cell (NSC) development pathways (such as Notch signaling). With Tuj1 and MAP2 expression raised 19-fold and 13.2-fold, respectively, this plasma–nanomedicine system boosted NSC differentiation into dopaminergic neurons from 9.3% to 70.1%. Treatment significantly enhanced motor function and restored dopaminergic neurons in 6-OHDA PD mice models.

Overall, two key mechanisms—the removal of abnormal protein aggregates and the stimulation of neuronal regeneration—have shown plasma–nanomedicine to be highly effective in NDDs. These methods offer a promising path for targeted therapy intervention by improving behavioral outcomes and pathological indicators. Although plasma–nanomedicines have shown notable benefits in the treatment of stroke, brain tumors, and NDDs, there are still a lot of research gaps. Firstly, common brain diseases like glioma, stroke, AD, and PD are the primary focus of contemporary research. There is an urgent need to expand the application scenarios and investigate different forms of brain diseases, such as cerebral vascular malformations, infectious brain diseases, genetic brain diseases, etc. In addition, this technology has not yet entered the field of mental disorders (like depression and schizophrenia), primarily because of the multi-target nature of the action of mental disorders (like abnormal neural circuits and monoamine neurotransmitter systems), the complexity of the pathological mechanism network (involving multiple factors like genetics, environment, and neuroimmunity), and the subjectivity of efficacy evaluation (mainly relying on scale scores and lacking objective molecular markers). It is necessary to design targeted research plans in combination with the particularity of mental diseases. Finally, the majority of studies are still in the cellular and animal experiment stage, lacking validation through large animal models (like non-human primates). Important problems like the long-term in vivo safety of nanomedicine (like metal ion accumulation toxicity and immunogenicity) and the standardization of plasma treatment dosages (like active particle concentration and irradiation time) remain unresolved. Establishing technical standards and assessment methods that satisfy clinical requirements is therefore essential. Because of this, currently, the field of plasma–nanomedicine technology still faces numerous significant theoretical and technical obstacles.

## 5. The Current Clinical Application Status of Plasma–Nanomedicine

Nanomedicine is a comprehensive interdisciplinary subject that applies the principles and methods of nanotechnology to the medical field, involving numerous fields such as medicine, materials science, physics, chemistry, biology, and quantum mechanics. Over the past three decades, a variety of nanomedicines have achieved clinical approval and have been widely applied in fields such as oncology, infections, cardiovascular diseases, rare diseases, and nucleic acid drug delivery, such as liposome drugs like pegylated liposomes doxorubicin (Doxil/Caelyx), as well as nucleic acid drugs delivered by lipid nanoparticles (LNP), etc. [96]. Compared with traditional preparation, nanomedicine can enhance brain accessibility and cross-BBB potential through surface modifications (such as ligand-mediated transport, adsorption-mediated transport, and vector-mediated transport), charge regulation, and biomimetic strategies (such as cell membrane encapsulation and exosome-like carriers), thereby increasing local effective concentration and improving the therapeutic window [97]. Secondly, nanomedicine can also reduce drug toxicity and improve the pharmacokinetic and administration experience by prolonging cycle time, reducing non-targeted distribution, and using sustained-release or controlled-release methods [98]. In conclusion, nanomedicine has a promising future in improving pharmacokinetics, reducing toxicity, and achieving tissue targeting.

CAP has been widely applied in antibacterial treatment, healing promotion, and tumor adjuvant therapy due to its high-energy activity and the generation of strong oxidizing particles [99]. In a randomized, placebo-controlled clinical trial, CAP significantly enhanced the reduction in wound area within the short-term treatment window for diabetic foot ulcers (DFU) (the remaining area at the end of treatment was 30.5% vs. 55.2%, *p* = 0.03), and accelerated the attainment of the early clinically significant threshold for area reduction (*p* ≤ 0.01) [100]. However, CAP research on the CNS is still relatively limited, especially in the field of neuropsychiatric diseases, and most of the existing work is still in the stage of mechanism exploration and proof-of-concept [101]. However, the research foundation of nanomedicines for brain treatment has formed a variety of strategic systems, including receptor-mediated transcellular transport, biomimetic/cell membrane engineering, lesion microenvironment response, local administration, and multimodal diagnosis and treatment integration [102]. Based on this, combining the tunable redox biological effects of CAP with the cross-barrier ability, spatiotemporal controlled release, and local enrichment advantages of the nanodelivery system is expected to achieve stronger local therapeutic effects and more controllable safety windows in the treatment of brain diseases, providing a direction worthy of attention for subsequent translational research.

## 6. Prospects

Plasma–nanomedicine synergistic therapy, as an innovative interdisciplinary treatment approach, has demonstrated significant value in the diagnosis and treatment of brain diseases by integrating the active regulatory advantages of plasma technology with the targeted delivery properties of nanomedicine. Mechanically, ROS produced by plasma can damage the structural integrity of the tumor cell membrane, thereby establishing an effective drug delivery channel. These channels increase the concentration of intracellular drug accumulation and improve therapeutic targeting. The continuous accumulation of ROS directly causes apoptosis of tumor cells, establishing a synergistic effect of “physical regulation + biochemical removal”. More importantly, this synergistic approach can drastically reduce the medication dosage or plasma intensity necessary to achieve the effect of a single therapy while guaranteeing therapeutic effectiveness. Therefore, it can minimize oxidative damage to normal nerve cells and vascular endothelial cells and effectively increase the biological safety of treatment. In the domain of drug delivery, plasma has the potential to transiently and reversibly regulate the permeability of the BBB, thus eliminating crucial obstacles for the penetration of nanomedicine into deep-seated brain tissues. In the field of drug delivery, plasma can transiently and reversibly regulate the permeability of the BBB, thereby eliminating important barriers for nanomedicine to penetrate deep brain tissue. At the same time, nanomedicine can further improve inter-barrier transport efficiency and lesion accumulation capacity through precise size optimization and targeted molecular modification. This innovation provides a potential solution to the key challenge that standard medications often fail to penetrate the BBB or accurately target brain lesions for refractory brain disorders, like brain tumors and deep cerebral vascular problems. Notably, plasma technology exhibits characteristics such as non-invasive intervention, controllable side effects, and high clinical safety, which differ from the features of traditional brain disease treatment technologies like Gamma Knife and proton therapy, such as invasive procedures and protracted postoperative recovery periods. These advantages give a firm basis for the clinical translation of this collaborative therapy method.

However, there are no proven technical frameworks or clinical application paradigms, and research on plasma and nanomedicine synergistic therapy for brain illnesses is still in the fundamental investigation stage. The following critical scientific challenges and technological bottlenecks urgently require breakthroughs:

1. Systematic theoretical research is insufficient, and the clarification of the core mechanism is limited. Current academic research has not fully explored three key aspects: the interaction mechanisms of plasma–nanomedicines in the complex brain microenvironment, the synergistic modes between plasma-derived ROS/RNS and nanomedicines, and the regulatory effects of this system on glial cells, neurons, and the BBB. Most existing theories are based on in vitro cell studies or simple animal models. These theories do not adequately explain how processes work within the complex environment of living organisms. As a result, the development of therapies lacks precise theoretical foundations.
2. Inadequate support for precise therapy and delayed research and development of specialist medical equipment. The execution of plasma–nanomedicine combination therapy needs specific apparatus. Nevertheless, the majority of current devices are general-purpose instruments repurposed from laboratories. These systems are confronted with difficulties such as sub-optimal management of plasma parameters, inferior efficacy in brain targeting, and the absence of real-time therapy feedback. The dearth of specialized and integrated equipment that complies with clinical application standards has emerged as a crucial bottleneck impeding technological translation.
3. Inadequate targeted disease research characterized by limited scope and depth. Present studies primarily concentrate on a handful of brain disorders, such as brain tumors, whereas investigations into high-prevalence conditions, including ischemic stroke, traumatic brain injury, PD, and AD, are still scarce. Furthermore, the majority of research still remains in the preliminary intervention phase of disease models. Meanwhile, most studies are still at the preliminary intervention stage of disease models. The treatment adaptability of different disease stages and pathological subtypes, as well as the safety and effectiveness of long-term treatment, have not been studied deeply, so it is difficult to form a targeted treatment plan.
4. Restricted application scenarios and unexploited potential in high-value domains. Currently, research on plasma–nanomedicine synergistic technology is predominantly focused on simple applications like antibacterial therapy. However, related research is still in its early stages in intricate and clinically important domains like the treatment of brain diseases. The technical promise has yet to be completely exploited, failing to fulfill the different therapeutic needs of brain illnesses.
5. The synergistic efficacy is limited by the delayed development of specialized nanomedicines. Nanomedicines directly affect the effectiveness of combination therapies as carriers and tailored delivery mechanisms for plasma-active components. Nevertheless, nanomedicines painstakingly developed for brain illnesses are still in short supply. This circumstance makes it laborious to accomplish accurate delivery and effective release of plasma active components, hence substantially restricting the synergistic therapeutic benefits.

To tackle these challenges, future studies ought to concentrate on making advancements in the following areas:

1. Expanding the study of basic mechanisms and creating a methodical theoretical framework. Focus on the interaction mechanisms at the interface between plasma-generated ROS and RNS and nanomedicines, and measure how they influence the regulation of cellular signaling pathways. Investigate how redox signaling pathways, such as those involving superoxide dismutase and glutathione peroxidase, contribute to neuroprotection facilitated by plasma. Elucidate how elements in the brain microenvironment (e.g., pH, enzyme concentrations, hypoxic conditions) affect synergistic therapeutic results, thereby establishing theoretical underpinnings for optimizing treatment regimens.
2. Promoting the research and development of specialized equipment for precise integration. Create low-energy plasma probes that can pass through the skull, and combine magnetic resonance imaging (MRI) with optical imaging techniques to develop a multimodal monitoring system for real-time visualization and dynamic treatment management. Enhance electrode materials, such as nano-conductive polymers, and refine structural designs to increase the stability and consistency of plasma output. Develop compact and portable devices to meet a variety of clinical needs.
3. Expanding the field of disease research and encouraging the investigation of tailored treatments. On the one hand, broaden the range of illnesses being studied, focusing on cooperative treatment research for neurodegenerative diseases, including Alzheimer’s and Parkinson’s, as well as ischemic stroke, in order to clarify the compatibility of various disease pathologies and synergistic treatment approaches. On the other hand, in order to provide a solid foundation for the development of customized treatment plans, stratified studies should be conducted to investigate the effects of treatment timing, dosage, and duration on the therapeutic effect of the same disease at various stages and pathological subtypes. Additionally, long-term animal experiments should be used to systematically assess the safety and long-term effects of the treatment.
4. Increasing the range of technologies’ applications and revealing high-value therapeutic potential. By concentrating on intricate situations like focused brain disease treatment, this innovative method breaks the constraints of traditional sterilization. It achieves an effective “targeted delivery + synergistic killing” therapeutic strategy by precisely delivering plasma-based active components to affected locations by utilizing the targeting power of nanomedicines. This technology is being employed to integrate the diagnosis and treatment of brain disorders by integrating the imaging capabilities of nano-probes with the therapeutic effects of plasma, providing a closed-loop system for “diagnosis-treatment-monitoring.”
5. Creation of targeted nanomedicines to improve combined treatment effectiveness. In view of the pathological aspects and treatment requirements of brain illnesses, we create plasma-responsive nanocarriers (e.g., ROS-sensitive nanovesicles) to enable adaptive and precise drug delivery. Surface modification tactics of metal and carbon-based nanomaterials are tuned to enhance their blood–brain barrier penetration efficiency and biosafety. Notably, plasma has the capacity to activate water molecules within nanomedicines, bringing an extra level of physical regulation to the treatment. Because of their improved compatibility with aqueous systems, high drug-loading capacity, and precise responsiveness, high-water-content nanomedicines, such as liposomes and porous materials, have emerged as attractive carrier alternatives. Their elevated water content gives advantages in formulation creation, permitting the direct synthesis of bioactive molecules via plasma for illness therapy. Liposomes’ biofilm-mimicking properties and porous materials’ huge specific surface area allow them to simultaneously encapsulate lipophilic and water-soluble medications, realizing a “single-carrier, multi-drug” synergistic therapeutic paradigm.

## 7. Conclusions

As a new area of study, plasma–nanomedicine presents both opportunities and obstacles. By fostering collaboration among researchers in materials science, biomedical engineering, and plasma physics, technological advancements can be achieved, key challenges overcome, therapeutic approaches diversified, and the effectiveness and safety of treatments enhanced. It is expected that these initiatives may promote the development of brain disorder treatments. Ultimately, these efforts may contribute to medical innovation in neuropsychiatric fields, improve the quality of clinical care for patients, and provide relevant support for human health and well-being.

## Figures and Tables

**Table 1:** Examples of plasma–nanomedicine therapy for brain diseases.

Nanoparticles	Therapeutic Agents	Size	Disease	Mechanism of Therapy	Effect of Therapy (Compared with Single-Drug Treatment)	Ref.
AuQDs	/	4–5 nm	Glioblastoma	Coordinately enhance ROS/H_2_O_2_ levels; Activate the Fas/TRAIL-mediated death receptor pathway; Enhance the expression of E-cadherin.	The mortality rate of tumor cells has increased by 1.56 to 1.88 times.	[74]
AuNPs	/	20 nm	Glioblastoma	CAP induces RONS; ATP-dependent endocytosis stimulation; Increased cytotoxicity.	The cytotoxicity increased significantly by 25 times.	[75]
PEG-coated gold nanoparticles (GNPs)	AuNPs	27–33 nm	Glioblastoma	Synergistic effect increases the level of ROS; Induction of p53-mediated cell apoptosis; Inhibition of EMT and maintenance of cancer stem cells.	The tumor weight reduction rate reached 50%; Combined treatment resulted in a 33% cell death rate.	[76]
PVA-AgNPs	AgNPs	10.68 ± 1.98 nm	Glioblastoma	Promote the cellular uptake of silver nanoparticles; Increase the generation of ROS; Oxidative stress induces cell death.	The cytotoxicity of PVA-AgNPs + CAP (25 s) increased by 25 s; The cytotoxicity of PVA-AgNPs + CAP (60 s) increased by 473 s.	[77]
Micelle-MB	MB (methylene blue)	7.39 nm	Glioblastoma	Double ROS superposition (CAP directly produces ROS + MB photodynamically produces ROS)	The cytotoxicity has increased by approximately 1.53 times; The generation volume of ROS has increased by approximately 1.2 to 1.5 times.	[78]
anti-EphA3-TMZ@GNPs	Temozolomide (TMZ)	45.88 ± 1.9 nm	Glioblastoma	GNPs plasma photothermal effect; Oxidative stress mediates p53 activation and MGMT inhibition; Reversing the drug resistance of glioma cells.	The tumor suppression rate was 88.32%; The survival time of the mice was increased by 1.64 times.	[79]
KLVFF@Au-CeO_2_ (K-CAC)	KLVFF peptide and CeO_2_ NPs	100 nm	Alzheimer’s disease	Relying on the near-infrared plasmonic effect of Au NRs; Promote the penetration of the blood–brain barrier and the targeted binding of Aβ; Synergistic elimination of ROS and degradation of Aβ.	The permeability of the blood–brain barrier has increased fourfold, ROS has been cleared, and Aβ has been degraded and inhibited.	[58]
Cu_2−x_Se	/	14.7 nm	Parkinson’s disease	Non-thermal plasma effect-driven; The CaMK/CREB pathway mediates; Regulation of neural differentiation.	The rate at which neural stem cells differentiate into neurons has increased by about 7.5-fold.	[67]

## Data Availability

No new data were created in this study. Permission for re-use of previously published figures has been obtained.

## Abbreviations

The following abbreviations are used in this manuscript:

CAP	Cold atmospheric plasma
AD	Alzheimer’s disease
PD	Parkinson’s disease
WHO	World Health Organization
ROS	Reactive oxygen species
RNS	Reactive nitrogen species
RONS	Reactive oxygen/nitrogen species
BBB	Blood–brain barrier
Aβ	β-amyloid
CN	Central nervous system
RMT	Receptor-mediated transcytosis
MPS	Mononuclear phagocyte system
PAW	Plasma-activated water
^•^OH	Hydroxyl radicals
O_2_^•−^	Superoxide anions
^1^O_2_	Singlet oxygen
O_3_	Ozone
H_2_O_2_	Hydrogen peroxide
NO^•^	Nitric oxide
NO_2_^•^	Nitrous oxide
NO_3_^•^	Nitrate
APNP	Atmospheric pressure plasma nanoparticles
APPJ	Atmospheric pressure plasma jet
OGD	Oxygen–glucose deprivation
cGMP	Cyclic guanosine monophosphate
PKG	Protein kinase G
DBD	Dielectric barrier discharge
MCAO	Middle cerebral artery occlusion
PLTV	Platelet vesicle
IO	Iron oxide
MB	Methylene blue
LSPR	Local surface plasmon resonance
Au NRs	Gold nanorods
AuNPs	Gold nanoparticles
CeO_2_ NPs	Cerium oxide nanoparticles
NIR	Under near-infrared
VPNS	Violet phosphorus nanosheets
PLGA	Poly L-glutamic acid
PEG	Polyethylene glycol
PCL	Polycaprolactone
PGA	Polyglycolic acid
EPR	Enhanced permeability and retention
GNPs	PEG-coated gold nanoparticles
CS NPs	Cu_2−x_Se nanoparticles
NSCs	Neural stem cells
NPD@M	MXene-based biomimetic plasma nanoplatform
DOX	Doxorubicin
TGFβ	Transforming growth factor beta
TRKI	TGFβ receptor kinase inhibitor
TLNs	TRKI-loaded lipid nanoparticles
HA	Hyaluronic acid
CHG	CAP-induced HA-based hydrogel
HAT	Hyaluronic acid–tyramine
ICD	Immunogenic cell death
PVA	Polyvinyl alcohol
AgNPs	Silver nanoparticles
KLVFF	Pentapeptide fragment corresponding to the Aβ16–20 region
KACA	KLVFF@Au-CeO_2_
GBM	Glioblastoma multiforme
TMZ	Temozolomide
CME	Clathrin-mediated endocytosis
NDDs	Neurodegenerative diseases
ALS	Amyotrophic lateral sclerosis
6-OHDA	6-hydroxydopamine
SOD	Superoxide dismutase
GPx	Glutathione peroxidase
MRI	Magnetic Resonance Imaging
TEM	Transmission Electron Microscopy
Nrf2	E2-related factor 2
